# Exploring Costimulatory Blockade-Based Immunologic Strategies in Transplantation: Are They a Promising Immunomodulatory Approach for Organ and Vascularized Composite Allotransplantation?

**DOI:** 10.3390/jpm14030322

**Published:** 2024-03-20

**Authors:** Andreea Grosu-Bularda, Florin-Vlad Hodea, Dragos Zamfirescu, Alexandru Stoian, Răzvan Nicolae Teodoreanu, Ioan Lascăr, Cristian Sorin Hariga

**Affiliations:** 1 Department 11, Discipline Plastic and Reconstructive Surgery, Bucharest Clinical Emergency Hospital, University of Medicine and Pharmacy Carol Davila, 050474 Bucharest, Romania; andreea.grosu-bularda@umfcd.ro (A.G.-B.); razvan.teodoreanu@umfcd.ro (R.N.T.); ioan.lascar@umfcd.ro (I.L.); cristian.hariga@umfcd.ro (C.S.H.); 2 Clinic of Plastic Surgery, Aesthetic and Reconstructive Microsurgery, Emergency Clinical Hospital Bucharest, 050474 Bucharest, Romania; 3 Zetta Hospital, 050474 Bucharest, Romaniaalecsandrustoian@yahoo.com (A.S.)

**Keywords:** costimulatory molecules, immunology, allotransplantation, transplant, vascularized composite allografts

## Abstract

The field of transplantation, including the specialized area of vascularized composite allotransplantation (VCA), has been transformed since the first hand transplant in 1998. The major challenge in VCA comes from the need for life-long immunosuppressive therapy due to its non-vital nature and a high rate of systemic complications. Ongoing research is focused on immunosuppressive therapeutic strategies to avoid toxicity and promote donor-specific tolerance. This includes studying the balance between tolerance and effector mechanisms in immune modulation, particularly the role of costimulatory signals in T lymphocyte activation. Costimulatory signals during T cell activation can have either stimulatory or inhibitory effects. Interfering with T cell activation through costimulation blockade strategies shows potential in avoiding rejection and prolonging the survival of transplanted organs. This review paper aims to summarize current data on the immunologic role of costimulatory blockade in the field of transplantation. It focuses on strategies that can be applied in vascularized composite allotransplantation, offering insights into novel methods for enhancing the success and safety of these procedures.

## 1. Introduction

The first successful renal transplant was performed in 1954 by Dr. Joseph Murray. Since then, the usage of human organs for transplants has gradually and constantly increased over the last decades, giving birth to an astounding field of medicine with remarkable therapeutic options and excellent long-term results. Transplants are currently performed in dedicated clinical centers all around the world, with standardized patient selection, surgical protocols, pre-surgery and follow-up management plans, as well as immunosuppressant therapy [1,2].

A new therapeutic option gained traction in recent years: reconstructive transplant surgery, which uses vascularized composite allografts for complex cases with severe defects that involve multiple layers of functional tissue, impossible to repair using conventional surgical techniques [3,4].

Vascularized composite allografts have been performed for life-enhancing indications in a selected group of patients under institutional protocols. The first hand transplantation was performed in Lyon, France, in the year of 1998. This historical cornerstone pushed the boundaries of research, resulting in the unexpected and rapid growth of this newly developed medical field. Numerous clinical procedures have been performed: upper limb, lower limb, larynx, uterus, penis, tendons, abdominal wall, nerves, face, tongue and scalp transplantations [3,5,6,7,8,9,10,11,12]. The main characteristic of composite allografts is their complexity, as determined by their histological heterogenicity, comprising multiple tissue types (skin, fat, muscles, nerves, lymphatic ganglia, bone, bone marrow, cartilage and ligaments), each presenting a different grade of immunogenicity [13,14].

The current limits, after two decades of clinical research experience on vascularized composite grafts, are (with standardized indications and surgical protocols) lifelong immunosuppression for a non-vital procedure and the adverse reactions stemming from such treatment [3,6,15,16].

Currently, the main research direction in composite tissue transplant research is within the immunology domain. The aim at the moment is to minimize immunosuppression to a point at which both the allograft functionality is preserved and adverse reactions are avoided. The ultimate goal is to induce donor-specific immune tolerance which presents a lack of specific reaction towards the donor’s antigens, in the absence of an immunosuppressant therapy and an unhindered immune system, with appropriate response towards other types of antigens. The desired effect is represented by “true” and complete tolerance (also referred to as clinical operational tolerance), with full body acceptance of the donor’s allograft, without the need for immunosuppressant therapy; an ideal situation, difficult to achieve unreachable in a clinical situation. Partial tolerance is, on the other hand, a more realistic vision. It represents the possibility of inducing a degree of tolerance which allows diminishing of immunosuppressant doses and, as such, the chance of secondary adverse reaction onset. Therefore, vascularized composite allografts could be used extensively due to their highly reconstructive potential [17,18,19,20,21,22].

Mechanisms for inducing immunologic tolerance to self can be divided into two categories based on anatomic location: central tolerance and peripheral tolerance. The first mechanism, central tolerance, takes place in the primary lymphatic organs such as the bone marrow and thymus. The second mechanism, peripheral tolerance, takes place in secondary lymphatic structures such as the spleen and lymph nodes, or even the donor organ itself (which triggers an immunologic response). These mechanisms have not been widely experimented on, especially when attempting tolerance induction in humans. Even fewer attempts have been made in tolerance induction strategies in patients who have undergone transplantations [21,23,24,25].

## 2. Materials and Methods

This study was created as a review of the existing medical literature focusing on immune modulation strategies applicable to vascularized composite allotransplantation (VCA). The primary objective was to explore innovative approaches that enhance the effectiveness and safety of immunologic therapies in VCA. Data were drawn from medical databases such as PubMed and Google Scholar by conducting a search using a combination of keywords: VCA, transplantation, immunologic therapies, costimulatory molecules and costimulatory blockade. The data synthesis aims to provide a comprehensive overview of the evolution of the field and the current state of immunologic therapeutic strategies in VCA, identifying novel approaches that need further clinical validation.

## 3. Immunologic Strategies in VCA

### 3.1. T Cell Activation

T lymphocytes have a central role in the immune reaction to transplanted allografts. Therapeutic strategies that interfere with T cell activation are very promising in modulating the immune response, preventing rejection and increasing the survival of the allograft [26,27,28].

T cell activation follows three important stages:

The first step comprises antigen recognition of donor-derived peptides, in addition to antigens of MHC as expressed by the allograft, through the lymphocyte’s TCR. After this interaction, the T cell will receive its first stimulus through the fastest signaling pathway using CD4/CD8 [29,30].

The binding of CD28 situs on the T cell represents the second step in the activation of the lymphocytes. Specific ligands of CD28 are expressed by the APC of the donor, such as CD80 or CD86. Should this stimulus not occur, in the sole presence of antigen presentation, the T cell will undergo a change toward clonal anergic status [30].

Thirdly, after the complete relay of the second signal through co-stimulatory molecules, the cytokine secretion begins in the T cell (IL-2). Thus, the third activating signal is generated after the interaction between cytokines and TCR, resulting in T cell differentiation [30].

**Figure 1:**
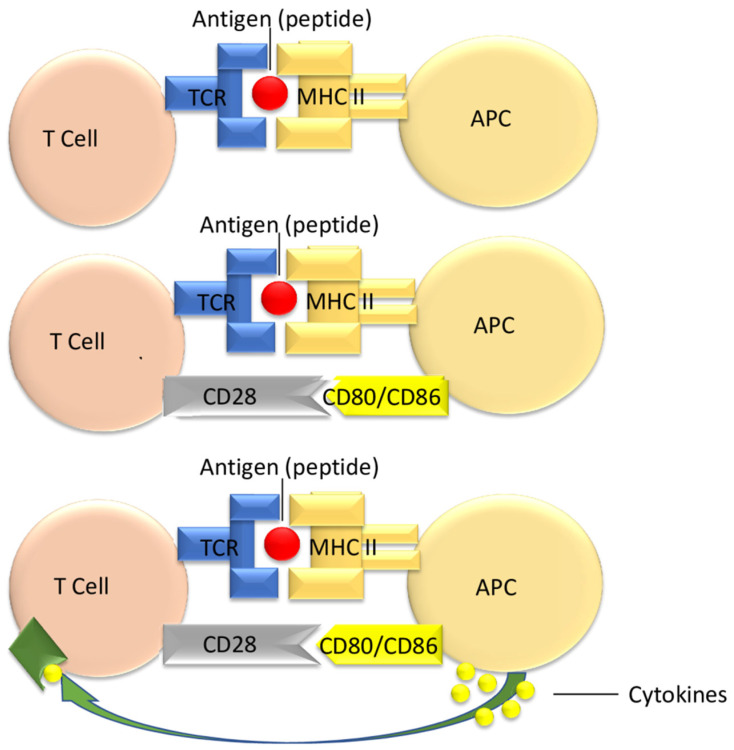
Adapted after Gutcher I (2007), under a Creative Commons Public Domain Mark 1.0 License [31].

Figure 1 depicts the three types of activating signal pathways for the T cell, of uttermost importance in the immune response leading to allograft transplant rejections [31].

Activation of T cells, especially the CD4+ subset, represents an essential timeframe in allograft rejection. The main mechanism of action pertaining to such activation is associated with pro-inflammatory cytokine discharge [30,32].

### 3.2. Targeting Costimulatory Pathways in Transplant Patients

T cell lymphocytes may become activated and determine an efficient immunologic response after receiving the activating signals beforehand. If the second signal needed for the activation of the co-stimulatory pathway does not take place, cells may not undergo activation, resulting in apoptosis, inactivation or becoming anergic [28,33]. Therefore, interfering with T cell activation through costimulation blockade strategies offers the perspective of prolonging allograft survival through the modulation of the immune response [34,35].

Based on their functional characteristics, costimulatory molecules can be divided into “positive”, which may enhance immune effective response, and “negative” costimulatory molecules, which may determine a decrease in the potency of the alloimmune reaction [35,36,37]. The classification of molecules is represented in Table 1.

### 3.3. The Immunoglobulin Superfamily

#### 3.3.1. The B7 Family

CD28 represents the main costimulatory receptor of the B7 family. It possesses two ligands expressed on the antigen-presenting cells (APC): B7.1 (CD80) and B7.2 (CD86). CD28 signals are essential for T cell activation, proliferation and survival, after the interaction between T cell and the APCs [50,51].

The cytotoxic T-lymphocyte-associated antigen 4 (CTLA-4), the first inhibitory molecule discovered, is a member of the immunoglobulin superfamily and presents an inhibitory inducing immunologic response, having the ability to bind to both B7-1 and B7-2 [52,53]. It is similar in structure to CD28, as seen on T cells in a resting state, and may be expressed only after cell activation. Both CD28 and CTLA4 can bind to CD80 and CD86 on APCs [54,55].

CTLA-4 presents a higher binding affinity for B7 molecules than CD28, acting therefore as a competitive antagonist, determining the inhibition of CD28-dependent T cell activation, cell cycle progression and IL-2 production [51,56].

CTLA-4 upregulation is dependent on CD28 activity. It has been noted that inhibition of CTLA-4 is more prominent after initiation of T cell activation and after constant signaling by CD28 [57].

Genetically engineered mice with deleted CTLA-4 genes were revealed to be more prone to developing severe lymphoproliferative diseases and premature death [58]. Autoimmune conditions are exacerbated by the administration of CTLA4-blocking monoclonal antibodies, which in turn induce T cell anergy as well [59].

Therefore, CTLA-4 can act as a competitive antagonist of CD28, while overall having a down-regulatory effect on the immune response. CTLA-4 is also essential for stopping T cell-mediated immune response through a feedback loop mechanism [60,61].

The induction of transplant tolerance may be obtained by selectively inhibiting the B7/CD28 pathway without interfering with that of B7/CTLA-4, thus down-regulating T cell response [57].

The strategy of direct CD28 blockade using monoclonal anti-CD28 antibodies showed agonistic undesirable side effects. Therefore, the fusion protein CTLA4-Ig—abatacept (consisting of two parts, the first being CTLA4 extracellular soluble domain and the second being an IgG1 heavy chain) was developed [35]. Abatacept (Orencia^®^, Bristol Myers Squibb, Princeton, NJ, USA), (CTLA4-Ig) acts as a competitive inhibitor for CD28 situs-binding proteins, causing T-cells to become anergic. Abatacept is currently an approved therapeutic strategy in rheumatoid arthritis and autoimmune disorders [62].

The ABA2 trial, a phase II study, explored the effectiveness and safety of abatacept in combination with standard treatments for preventing severe acute graft-versus-host disease (AGVHD) following unrelated donor hematopoietic cell transplants in patients with hematologic malignancies (divided into two groups: those receiving HLA-matched and those receiving HLA-mismatched transplants). The results indicated that abatacept effectively reduced severe AGVHD in both groups, with particularly striking improvements in the HLA-mismatched group. The drug was also associated with better survival rates free of severe AGVHD. However, it did not significantly impact the incidence of chronic GVHD. Overall, the study suggests abatacept as a promising addition to existing preventive strategies for AGVHD, potentially improving the outcomes in hematopoietic cell transplantation, especially in HLA-mismatched cases [63].

It also presents a favorable result in transplant therapy due to its favorable effect on the CD28-B7 pathway. This effect, however, is hindered by the simultaneous administration of cyclosporine, a calcineurin inhibitor and widely used immunosuppressant. While acute rejection was inherently avoided, chronic rejection represented a major concern when the two drugs were administered concomitantly [64].

Abatacept may be considered for early conversion therapy in transplant patients, as reported by Badell in a series of nine renal transplanted patients, in which calcineurin inhibitor immunosuppressant therapy was not indicated; all the patients of this study survived and presented good kidney allograft survival in the long-term follow-up [65].

Belatacept (LEA29Y) represents an alternative to abatacept, the difference being its higher affinity for target receptors and higher potency [41,66,67].

The literature regarding belatacept is widely based on solid organ transplantation research due to the common interest in this area and the plenitude of performed relevant clinical trials. In 2011, the Food and Drug Administration (FDA) approved belatacept (Nulojix^®^, Bristol Myers Squibb, Princeton, NJ, USA) for the prevention of acute rejection in adult recipients of kidney transplants [68,69,70,71].

The current recommendation for belatacept is to be administered alongside other immunosuppressants such as basiliximab induction, mycophenolate mofetil (MMF) or corticosteroid therapy [71].

In kidney transplant, belatacept may provide a sufficient immunosuppressive effect without any of the side effects of standard immunosuppressant therapy. Compared with calcineurin inhibitors, belatacept showed no difference in preventing acute rejection episodes, graft loss or death. However, it offered some advantages: better kidney function after transplant, less chronic kidney scarring, decreased rate of donor-specific antibodies development, improved blood pressure values, better lipid blood levels and lower incidence of diabetes onset [72,73,74].

A peculiar limit to belatacept administration poses for Epstein–Barr virus (EBV) sero-negative kidney transplant recipients. It was shown that after receiving high doses of belatacept, such patients showed a higher risk of developing post-transplant lymphoproliferative diseases (PTLD), predominantly PTLD of the central nervous system. After such clinical observations, belatacept may only be recommended for EBV sero-positive patients [71,75,76].

Belatacept seems to also be a promising agent in the therapeutic regimen administered after vascularized composite allotransplantation, with the advantage of reducing long-term complications, such as nephrotoxicity, in transplant recipients [74].

Cendales et al. reported a clinical case of a recipient of hand allotransplantation who presented recurrent episodes of acute rejection with consecutive alloantibodies increase. The patient also presented nephrotoxicity, following the administration of calcineurin inhibitors. In this case, maintenance immunosuppressive therapy (tacrolimus, mycophenolate mofetil and steroids) was replaced with a combination of belatacept, sirolimus and prednisone, which ensured an optimal clinical outcome, with good allograft evolution, no further rejection episodes and also improved renal function [77].

In another case of unilateral hand transplant from the same team, a 54-year-old man, received, after lymphocyte depletion, de novo-belatacept-based therapy with short-term calcineurin inhibitors administration. After rejection episodes in the first year post-transplant, successfully therapeutically reversed, the follow-up at twenty months after the hand allotransplant in this patient (beneficiating from a calcineurin-inhibitors-free immunosuppressive regimen) revealed good tolerance for belatacept, without further rejection episodes. De novo donor-specific antibodies were not detected, and protective immunity was maintained without viral reactivation (the patient’s viral profile was cytomegalovirus-positive and Epstein–Barr virus-positive) [78].

Belatacept therapy is indicated in the situation of antibody-mediated rejection, also presenting an advantage in preventing de novo alloantibody occurrence [79].

A clinical report from Innsbruck, Austria, included four male upper limb allotransplant recipients (three of them with bilateral transplant and one with unilateral transplant) who received belatacept therapy started within the range from 4 months to 13 years postoperatively. Despite the variable immunologic evolution of these patients, some observations were made: three of four patients showed a favorable response to belatacept conversion, allowing the reduction of calcineurin inhibitors doses (but not complete withdrawal), with good rejection control and negative tests for donor-specific antibodies. The fourth patient had a unilateral hand allotransplant and presented two episodes of cellular rejection, as well as two antibody-mediated rejections before belatacept was started six years post-transplant. Unfortunately, this patient had further unfavorable evolution with high levels of donor-specific antibodies, progressive rejection with vascular impairment and allograft necrosis necessitating amputation 7 years post-transplant [80]. A particular immunological aspect was observed in this patient: high levels of subset CD57+CD4+T cells (with CD 57+ representing 12% of the CD4+ cells population). The CD57+CD4+ subsets seem to be associated with therapeutic resistance to belatacept [74,80].

The Boston team reported a case of a face transplant patient who was converted to belatacept 14 months post-transplant due to severe standard immunosuppressant complications (tacrolimus, mycophenolate mofetil and prednisone scheme) with kidney impairment, neurological toxicity and persistent cytomegalovirus viremia. Consequent to belatacept conversion, renal function improved, and viral complication was controlled, but the occurrence of a rejection episode necessitated the reintroduction of a low-dose tacrolimus maintenance therapy, with good clinical outcome [81].

A study published by Krezdorn et al. reveals the high prevalence of kidney dysfunction in the beneficiaries of the vascularized composite allotransplants, emphasizing the mandatory role of the correct prevention and management of renal risk factors and also encouraging dose reduction of calcineurin inhibitors or, if possible, full withdrawal of these agents. Therefore, alternative immunosuppressive strategies including belatacept or mammalian target of rapamycin inhibitors may improve long-term outcomes, reducing nephrotoxicity [82].

In summary, belatacept has shown good results as a therapeutic strategy in kidney transplantation and seems to be a promising agent for clinical translation in the field of VCA. It may be introduced as de novo or in a delayed manner (even many years after transplantation) in VCA maintenance immunosuppressive therapy, with a considerable advantage in reducing calcineurin inhibitors’ side effects. The downside of belatacept therapy is that acute rejection episodes remain possible. A major indication for belatacept use is in cases with antibody-mediated rejection, belatacept being also efficient in the prevention of alloantibody appearance.

Further research is needed to establish a decision-making algorithm for the clinical use of belatacept in VCA patients, also exploring the immunologic profile of the patients and the determination of predictive markers (specific immune cells subsets expression) of the efficiency of resistance in belatacept therapy [74,79,80,83].

#### 3.3.2. The ICOS-B7h Pathway

ICOS (inducible costimulatory molecule), a member of the CD28 superfamily, is present after the activation of CD4+ and CD8+ T lymphocytes and is persistent in memory T lymphocytes and effector lymphocytes [35,84]. ICOS is expressed in both Th1 and Th2 cells, with a higher expression in Th2 cells [35]. Its ligand is represented by B7h (B7-H2, ICOS-L), expressed constitutively on antigen-presenting cells [84]. Signaling via the ICOS/B7h pathway increases the survival and proliferation of T cells and stimulates cytokine production and interactions between lymphocytic populations [35].

ICOS/ICOS-L blockade has been explored in experimental transplant models. Harada et al., using a vascularized cardiac allograft in a mouse model, demonstrated that the efficiency of ICOS/B7h costimulatory blockade (using monoclonal antibodies) in prolonging allograft survival was dependent on the timing of blockade regarding the dynamic of the immune response: the blocking effect was more potent in the differentiation/effector phase rather than the priming phase of the immune response to the allografts, encouraging ICOS-B7h blockade manipulation after priming of T lymphocytes [84].

ICOS pathway blockade determines allograft survival at a lesser level compared with CTLA4Ig and CD40 pathway blockade separately, but an interesting approach is represented by the combination of blockade of two pathways (co-blockade of ICOS/B7h and also CD28/B7 or CD40/CD40L), leading to synergistic effects in avoiding rejection of the allografts [35].

#### 3.3.3. The Programmed Death-1 (PD-1)/PD-1 Ligands

PD-1 (CD279) is one representative of the CD28 family. It can be found on myeloid and activated B and T cells. It possesses two ligands: PD-L1 and PD-L2. PD-L1(CD274, B7-H1) is a transmembrane glycoprotein, with the ability to block T cell activation. In order to accomplish such activation, it requires receptor ligation, namely by binding PD-1 with CD3 and/or CD28 [85]. PD-1 signals interfere with the activation of phosphatidylinositol-3-kinase (PI3K), which in turn is mediated by CD28. As a consequence, it inhibits the production of IL-2, determining an anergic state of the T cells. Cell apoptosis has also been shown to be induced by PD-1 [86].

While PD-L1’s role has been predominantly explored in cancer therapy, it holds considerable promise in managing both alloimmunity and autoimmunity. Existing research has largely concentrated on inhibiting PD-L1 to prevent immunosuppression, but augmenting PD-L1 activity could be a beneficial strategy for mitigating destructive immune responses. Further research is essential to fully comprehend the implications of increased PD-L1 expression and its availability in treating GVHD and autoimmune diseases, and in the context of organ transplantation, potentially promoting immune tolerance [87,88]. Studies indicate that enhanced PD-L1 expression in GVHD models can improve survival rates and decrease the secretion of pro-inflammatory cytokines. Similarly, in the context of organ transplantation, activating PD-L1 has been associated with extended graft survival and lower rejection rates. In models of autoimmune diseases, elevated PD-L1 expression has been linked to slowed disease progression and reduced tissue damage [87].

### 3.4. TNF Family Members

#### 3.4.1. CD40/CD40L(CD154) Pathway

In addition to CD28/B7, the CD40/CD154 pathway represents a second important pathway targeted in the transplantation field for the modulation of the immune response [35].

CD40 represents a member of the tumor necrosis factor-TNF receptor superfamily. It is widely expressed in B cells, macrophages, dendritic cells, thymus epithelial cells, endothelial cells and fibroblasts. Its ligand, CD154 (CD40L), is expressed in activated T cells and NK cells [89].

CD40/CD154 pathway is highly cardinal for activation of B and dendritic cells leading to increased secretion of humoral molecules such as IL-1, TNF-α and IL-12. The pathway’s effect on endothelial cells is to increase the secretion of monocyte chemotactic factors. CD40/CD40L also activates the antigen-presenting cells, which determines indirectly T cell activation [36]. Singular inhibition or combined inhibition with CTLA4-Ig of this pathway, on murine models, increases the tolerance of MHC-mismatched skin and cardiac grafts. Anti-CD154 molecules administered either alone or in addition to CTLA4-Ig molecules prevented acute renal allograft rejection in non-human primates [90,91].

Early literature already proved that allograft survival is prolonged by the administration of monoclonal antibodies directed against CD40L [92]. The occurrence of allograft vasculopathy in a murine aortic transplantation model is avoided by costimulatory inhibition (CD28-CD40L). In turn, it co-actively increases allogeneic murine skin and cardiac allograft survival [93,94].

Encouraged by very good results in animal models, in the early 2000s, translation to the clinical use of CD40/CD154 therapeutic blockade (using humanized or chimeric anti-CD154 monoclonal antibodies) was performed, but, unfortunately, the occurrence of thromboembolic complications precluded further development [95,96]. The use of anti-CD154 monoclonal antibodies can cause thrombotic effects through binding to CD154 developed by activated platelets [96,97]. In preclinical models, the development of thrombotic side effects was diminished through different strategies such as prophylactic peri-operative administration of anticoagulants(heparin) and aspirin or reducing the course of the administration of anti-CD154 monoclonal antibodies [96].

It was shown that a portion of the anti-CD154 monoclonal antibodies that binds to platelets (to their Fc receptor CD32A), contributing to thromboses, is the Fc domain; therefore, disabling the functions mediated through this domain may help avoid platelet aggregation and activation [96].

Another variant explored in order to avoid unwanted thromboembolic events is targeting the CD40/CD154 pathway using monoclonal antibodies directed to the CD40 component, with promising results in preclinical models of organ transplantation including large animals such as monkey models [96].

Recently, Harland and co. published a clinical report on using bleselumab (ASKP1240) therapy, an anti-CD40 monoclonal antibody for de novo administration in renal transplant recipients over a period of three years after transplantation. Bleselumab blocks the interaction between the CD40 receptor and its ligand CD 154, avoiding both cellular and humoral immune responses. There was a phase IIa, open-label, randomized study evaluating the bleselumab therapy safety and efficacy in preventing biopsy-proven acute rejection when combined with immediate-release tacrolimus or mycophenolate mofetil in renal transplant recipients. The results were compared with standard immunosuppressive therapy (immediate-release tacrolimus + mycophenolate mofetil). All patients also received corticosteroid therapy in low doses for 3 years post-transplant. This study concluded that the association of bleselumab and tacrolimus showed noninferiority compared with the standard of care for rejection prevention at 6 months and 3 years post-transplant; the failure of efficacy was higher in the bleselumab +mycophenolate mofetil group compared with the standard of care, while bleselumab and tacrolimus exhibited efficacy failure incidence similar to the standard of the care group. Therefore, bleselumab presents a good risk–benefit ratio for renal allotransplant recipients [98].

#### 3.4.2. The OX40 and OX40L Pathway

Another member of the tumor necrosis factor receptor family is represented by OX40. Its main roles are increasing the survival and maintaining the homeostasis of effector T cells, with a preferential effect on CD4+ T cells, both in transplants and immunogenic conditions [99,100,101,102].

In murine models, inhibiting the OX40–OX40L pathway determines a decreased immune response. Consequently, it diminishes the severity of different inflammatory or autoimmune disorders and also attenuates T-cell-mediated rejection [100,103,104].

Pertaining to the clinical literature to date, few research areas of the OX40 pathway are present. One of them is oxelumab, a human OX40L-targeting mAb, evaluated in a Phase II trial for the treatment of asthma. Another one is in that of the oncological domain. Immunogenic stimulation of this pathway, in advanced cancer patients, proved that circulating leucocytes (as seen in patients with melanoma) had had their antitumor properties changed, in addition to the immune regulation of intra-tumor regulatory cells. In patients with prostate cancer, beneficial anti-tumor effects were also reported [99,105].

In the transplant field, OX40/OX40L pathway blockade does not have a critical role in allograft survival (OX 40 not having significant importance for primary T cell responses), but plays a synergistic role with CD 28 or/end CD 154 blockade, increasing allograft survival. An important aspect is OX40’s implication for the outliving of activated T lymphocytes and the generation of memory T cells. Therefore, OX40/OX40L pathway blockade could address the memory cells resistant to other costimulation blockade strategies, this cell population representing an important problem for transplant recipients [35].

#### 3.4.3. GITR/GITRL Pathway

Glucocorticoid-induced tumor necrosis factor family-related receptor is present after the activation of CD4+ and CD8+ T cells on their surface and is constitutively expressed by regulatory T cells (T-regs) [35,106]. Through the GITR-GITRL pathway, the antigen-presenting cells increase the adaptive immune response to alloantigens by regulatory T cells counter-regulation and the costimulation of effector T cells [106]. GITR/GITRL costimulation determines T cell proliferation and stimulates cytokine production [35].

In an animal model (transgenic mice) of skin allograft transplantation, the blockade of GITR-GITRL through AITRL-Fc (a recombinant molecule that binds GITR) resulted in long-term acceptance of the skin allografts, through a T-regulatory-cell-dependent mechanism [106].

#### 3.4.4. 4-1BB/4-1BBL Pathway

4-1BB (CD137) pertains also to the TNF family, being expressed by T cells upon activation. Its correspondent ligand 4-1BBL appears on mature dendritic cells, macrophages and activated B lymphocytes. Signaling through the 4-1BB/4-1BBL pathway determines the activation, differentiation and survival of T lymphocytes and has an important role in the rejection of the transplanted grafts mediated by CD8+ T cells. The blockade of the 4-1BB costimulatory pathway determines allograft survival for intestinal and cardiac experimental transplant models, while for skin allografts rejection persists [35].

### 3.5. TIM Family

Discovered in 2001, T cell immunoglobulin (Ig) and mucin domain (TIM) molecules pertain to type I transmembrane glycoprotein family and seem to have an important role in immune response regulation [35,46]. In mice, there are eight genes of the TIM family, while in humans, only three are preserved: TIM-1, TIM-3 and TIM-4 [46].

The TIM molecules ensure a functional substrate that influences the activation and differentiation of T cells with a role in autoimmunity and allergic reaction, with proven immunological involvement in allotransplantation experimental models [35,46]. TIM molecules also determine T cell apoptosis, promote tolerance and increase the antigen-presenting cells’ ability to remove the apoptotic cells [35,107].

Experimental studies have been conducted on TIM-1 and TIM-3 involvement in transplant immunity, and there are also ongoing data regarding their role in human allotransplantation fields [46].

#### 3.5.1. TIM-1 Signaling Pathways

TIM-1 molecule is expressed preferentially on T helper 2 lymphocytes and is up-regulated consequently through T lymphocyte receptor stimulation. It is not primarily expressed in CD4+ T cells [46]. TIM-1 can bind to different ligands including TIM-4, phosphatidylserine, Igλ and TIM-1 itself [35].

TIM-4 is not expressed in T lymphocytes, but it appears constitutively in antigen-presenting cells including macrophages and dendritic cells [35,46]. TIM-4 seems to present bimodal behavior based on the activation status of T lymphocytes: it inhibits the activation of a naïve T cell via a TIM 1-independent pathway and induces a positive regulation for T cells which are activated; TIM-4–TIM-1 represents a costimulatory pathway for T cell activation [47,108].

Costimulatory pathways involving TIM-1 determine the survival, proliferation and cytokine production of T lymphocytes [35]. In murine experimental cardiac and pancreatic islet allotransplantation models, TIM-1 is an important immunomodulation player, involved in T lymphocytes effector/regulation balance [46]. Based on promising experimental results, targeting the TIM-1 pathways constitutes a field worth exploring, aiming to promote clinical therapeutic strategies that may overcome resistance to transplant tolerance [48].

#### 3.5.2. TIM 3 Signaling Pathways

TIM 3 represents a molecule widely expressed in the cells of the innate immune system such as mastocytes, macrophages and dendritic cells, but also T cells such as Th1 and Th17 (but specifically not in quiescent T cells or Th2 cells). Galectin 9, as seen expressed on the surface of naïve CD4+ T cells and Tregs, represents a binding situs for TIM 3. Its activation may induce a down-regulatory effect. Cell death is induced by the inhibition of Th1 as a result of TIM3 attaching to Galectin 9. Another effect of this linkage between the aforementioned molecules is the in vitro inhibition of Th17 differentiation [35,109].

A hinted effect of TIM3 signaling throughout the medical literature is a major regulation of allograft tolerance, due to the negative modulation of the T cell response in such cases [35].

### 3.6. Adhesion Molecule Blockade

#### 3.6.1. LFA-1 Pathway

Adhesion molecule pathways are amongst other co-stimulatory pathways researched for their effects in transplantation and autoimmune diseases, with emphasis on the LFA-1 (lymphocyte function-associated antigen 1) pathway. This particular pathway can be accessed due to the expression of LFA-1 in both CD4+ and CD8+ T cells. The latter presents consistent expression on the cell surface coupled with persistence. The transmission of T cell receptor-mediated signals as well as inflammation are two processes in which LFA-1 is involved. During inflammation, LFA-1 binds to the ligand ICAM-intercellular adhesion molecule on endothelial cells, thus initiating the sequence of immune cell migration to the inflammatory area, with emphasis on activated T cells [35,36]. The antagonism of LFA-1 has been proven practical in individuals who possess an increased number of memory T cells; such individuals are highly sensitized patients, for example, those who underwent multiple transplantations or transfusions, or those who suffer from inflammatory conditions or autoimmune disorders [36,99,110].

A successful LFA-1 antagonist that has had remarkable success in treating psoriasis is represented by efalizumab. This molecule has gained traction and studies have emerged regarding its potential benefits in graft rejection prevention after organ transplantation [35,36].

Blockade of the LFA-1 pathway showed good results in experimental transplantation, prolonging the survival of cardiac and islet allografts in non-human primates [35].

This pathway was also explored in VCA experimental models; in an allotransplant hindlimb model in rats, a blockade of the LFA-1/ICAM-1 interaction was tested along with a short-term administration of immunosuppressive systemic therapy, with improved long-term survival of the allografts in animals receiving adhesion molecule blockade [111].

Efalizumab was translated to clinical trials of pancreatic islet and renal transplantation with encouraging results regarding efficacy in the prevention of allograft rejection. An interesting finding is that the association between the antagonists of the LFA-1 pathway and sirolimus (rapamycin, a macrolide immunosuppressant) increases T regulatory cell levels in transplant recipients and inhibits rejection [99,112].

Unfortunately, further use of efalizumab was ceased after three patients (out of 40,000) developed a John Cunningham polyomavirus-associated disease-progressive multifocal leukoencephalopathy, a rare but deadly disease [99].

Therefore, the future of LFA-1 antagonism in the transplantation field is uncertain, but further research developing safer compounds is justified due to the promising results shown by LFA-1 blockade strategies in preventing allograft rejection and avoiding side effects such as renal toxicity associated with the administration of calcineurin inhibitors immunosuppressants [99].

#### 3.6.2. LFA-3 Pathway

LFA-3 (lymphocyte function-associated antigen 3) and CD2 (T cell surface antigen CD2) are two other poignant adhesion molecules that take part in the co-stimulatory pathways, with high expression on T memory cells surface [99].

Alefacept is a recombinant LFA3/IgG1 fusion protein. Although initially used in psoriasis treatment, it has also been showing promising results in transplantation due to its ability to decrease the number of memory T cells [113,114].

In a primate animal model of kidney transplant, Weaver et al. proved that renal allograft survival can be prolonged and alloantibody formation can be delayed by the administration of a combination of LFA3-Ig with CTLA4-Ig and sirolimus. The importance of such data is that there is a promising alternative for further immunosuppressive treatment schemes that do not require the usage of T-cell-depletion drugs, calcineurin inhibitors and corticosteroids. Therefore, it might reduce adverse reactions associated with commonly used immunosuppressants [115].

So far, two Phase II clinical trials have been conducted, using alefacept as de novo therapy in renal allotransplantation. In the first, conducted in Europe, alefacept was added to standard immunosuppressive therapy and was compared to a placebo group, while in the second randomized study (conducted in the USA), the aim was alefacept administration with the reduction of tacrolimus or mycophenolate mofetil doses [113,114]. Those studies showed that alefacept induction therapy did not significantly reduce the rejection rate (confirmed by biopsies) six months after renal transplantation; in consequence, the Astellas pharmaceutical company ceased the development of alefacept for renal transplantation [114].

Further studies of the LFA-3/CD2 pathway are needed for a clear understanding of its biology in allotransplantation, bearing in mind that this therapeutic approach may present important benefits for patients with previous immune sensitization, in whom memory T cell inhibition is mandatory [99,114].

## 4. Discussion

The use of human organs for transplantation has steadily increased over the past decades, with transplant medicine experiencing remarkable development and demonstrating great therapeutic potential [116]. Organ transplantation is the definitive treatment and survival strategy for individuals with terminal renal, hepatic or cardiac failure, significantly enhancing their quality of life. For severe, irreversible liver, heart or lung conditions, transplantation remains the only viable therapeutic option. While dialysis can support those with renal failure, kidney transplantation has been proven to offer superior survival outcomes, establishing it as the preferred treatment. Additionally, pancreas transplantation substantially benefits diabetic patients, and hematopoietic bone marrow transplantation serves as the standard treatment for a variety of hematologic malignancies and disorders [116,117,118].

Currently, transplant interventions are successfully carried out in specialized clinics worldwide. Patient selection, surgical protocols, pre-operative and post-operative management, as well as immunosuppressive therapy have already been standardized [116]. The advancement of science in the transplantation of solid organs and the development of reconstructive surgical procedures have allowed for the development of reconstructive transplant surgery, implying the transplantation of vascularized composite allotransplants, such as the face, upper and lower limbs, the larynx, the uterus and the penis [3,5,11,12]. The main challenges in this field of transplantation are the immunological aspects, namely the presence in the structure of a VCA allograft of multiple types of tissues with different antigenic loads, including the skin, which is a highly antigenic tissue. VCAs are not life-saving procedures but allow for the restoration of those components of the human body lost due to accidents, congenital malformations and surgical excisions when conventional reconstructive surgery is not possible. Unlike solid organ transplants, recipients of vascularized composite allografts are usually healthy patients, except for severe tissue defects, with the ultimate goal of the transplant being to improve the patient’s quality of life [14,15,17,18,119,120,121].

Long-term survival of the patient in the field of organ transplantation depends on the immunosuppressive therapies and their toxic complications [122,123]. Organ rejection can be divided into two categories: acute and chronic. In both types of rejection, T cell and antibody mechanisms are involved [124]. Regimens of immunosuppressants are key in the prevention of rejection, ensuring longevity, but taking into consideration possible complications. Involved agents are comprised of calcineurin inhibitors (CNIs), mTOR inhibitors, antiproliferative agents, corticosteroids and costimulatory blocking molecules. CNIs, such as tacrolimus and cyclosporin, are efficient, potent agents but may cause nephrotoxicity, hypertension and diabetes mellitus. mTOR inhibitors may cause proteinuria and wound healing impairment. Antiproliferative agents such as mycophenolate mofetil (MMF) may have adverse effects such as bowel disturbances and the suppression of bone marrow. Corticosteroids may have side effects such as osteoporosis, obesity, diabetes and an increased risk of infections. Immunosuppressants have been documented to increase the risk of malignancy and opportunistic infection; therefore, regimens must be balanced to prevent both rejection and agent-induced complications. Therapies have improved over the last three decades, with, however, no one-size-fits-all strategy for transplanted patients, the choice of regimen protocol depending on the type of organ, expertise, patient compliance and the ability to afford additional expenses, as well as individual particularities [125,126,127,128].

**Figure 2:**
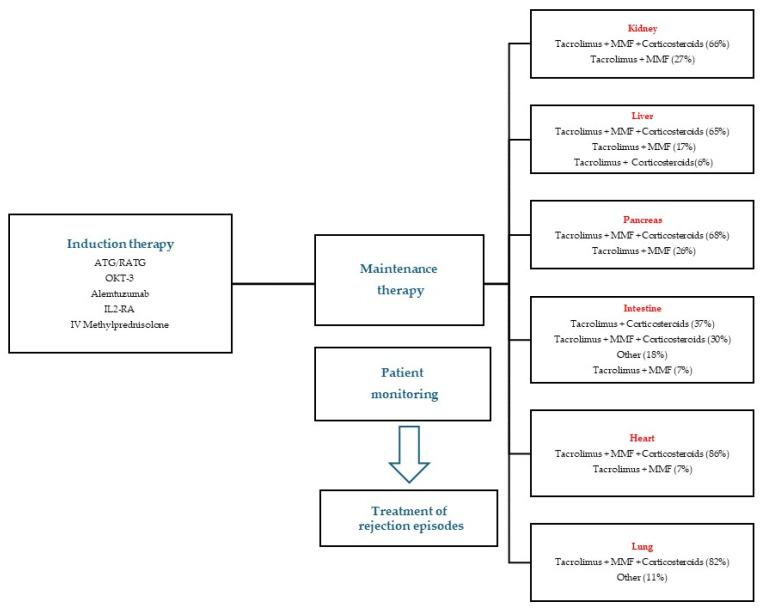
Therapeutic strategy in organ transplantation starting from induction, through maintenance, monitoring and rejection episode treatment. Maintenance organ-specific protocols are presented, with percentage description of protocol usage as main immunosuppressive regimen.

Treatment protocols consist of induction and maintenance immunosuppressive therapy. Transplant induction agents, including antithymocyte globulin (ATG), rabbit antithymocyte globulin (RATG), alemtuzumab, interleukin-2 receptor antagonists (IL2 RA), OKT3 (muromonab-CD3) and intravenous methylprednisolone administration are essential for conditioning the immune system to accept the transplanted organs. These agents work together to lower the risk of acute rejection, contributing to the overall success of the transplant procedure. Maintenance follows, using an array of agents that aim to diminish T cell activity, including the aforementioned calcineurin inhibitors, mTOR inhibitors and purine synthesis inhibitors. Steroid agents can be used in both stages for additional immunosuppressant activity. The patient is then monitored for signs and symptoms of rejection. In case rejection is suspected, empirical therapy may be initiated, usually by administering corticosteroids. If empirical therapy is successful, the patient continues monitoring as before, according to each transplant center protocol. However, a biopsy is the investigation that ensures a definitive diagnosis. If confirmed, treatment is adjusted based on severity, using high-dose corticosteroids in mild to moderate cases [125,126,127,128,129,130,131,132]. Anti-thymocyte globulin may be used as a rescue strategy in corticosteroid-resistant or severe rejections [133]. Figure 2 summarizes the immunosuppressive therapeutic strategies in organ transplantation [125,126,127,128,134,135,136,137,138,139,140].

Even more so in the case of vascularized composite transplants, which are not life-saving but have a functional indication, the immunosuppressive treatment must be carefully selected and managed to minimize the occurrence of severe adverse reactions [15,121]. Having components with different antigenicity, it is important to understand well the dynamics of the immune response and to carefully monitor the allograft and promptly address episodes of acute rejection [29,30]. The skin component of most vascularized composite transplants allows visual monitoring of the allograft and the harvesting of tissue biopsies, enabling therapeutic intervention in the early stages in case of an immune response with the correct adjustment of immunosuppressive treatment. The presence of a bone component in the allograft brings hematopoietic bone marrow, whose cellular components have an immunomodulatory influence by inducing hematopoietic chimerism [15,17,18,19,141,142].

Regarding immunosuppressive agents used, the protocols in composite tissue transplants are similar to those in solid organ transplants, especially the regimens used for kidney, heart or pancreas transplants (the liver is better tolerated as an organ, requiring milder immunosuppressive regimens) [18,143].

Agents used in face and upper limb transplants can be seen in Table 2, with usage frequency (%) in each of the immunosuppressive stages [131].

Triple therapy with tacrolimus, MMF and steroids is the standard protocol for maintenance immunosuppression in VCA recipients. Some centers attempt to reduce steroid doses or even eliminate them from the therapeutic scheme. To avoid the adverse effects of calcineurin inhibitors, the trend is to include mTOR inhibitors in maintenance therapy as a replacement for tacrolimus. In transplants that also have a skin component, topical agents have been used to control mild rejection episodes, even without the need to increase the intensity of systemic immunosuppressive therapy. The topicals used are steroid compounds and tacrolimus [19,37,131,144]. In VCAs, acute rejection can be reversed if timely detection and correct treatment are initiated. Over 80% of cases have been reported to resolve after administering corticosteroid bolus in both face and upper limb transplants with acute rejection. If the issue is not resolved, the patient may benefit from increasing the dose of maintenance agents, the usage of anti-thymocyte globulins, basiliximab or alemtuzumab, or even the topical application of tacrolimus or dexamethasone [19].

Monitoring the effectiveness of immunosuppressive therapy in solid organ transplants is vital for maintaining graft health and reducing rejection and side effects risks. It involves clinical assessments, lab tests and imaging. Essential tests measure immunosuppressant blood levels to avoid under- or over-immunosuppression. Organ biopsies check for rejection or damage, while organ function is tracked using markers such as serum creatinine and GFR for kidneys, liver enzymes for livers and spirometry for lungs. Detecting donor-specific antibodies helps spot early antibody-mediated rejection. These methods allow for timely immunosuppressive therapy adjustments to enhance graft longevity and patient outcomes [145,146,147].

VCA monitoring requires detailed strategies to identify rejection signs and ensure transplant success. Unlike solid organ transplants, VCAs such as hands and faces pose challenges due to their mix of skin, muscle, bone and sometimes nerves. Monitoring includes physical exams for signs such as color changes, swelling and the appearance of lesions. Skin biopsies may be performed for early rejection detection, and functional and imaging studies for assessing tissue integrity. Blood tests for immunosuppressive drug levels help balance minimizing rejection risks and drug toxicity. Recent developments in biomarkers and molecular diagnostics offer more accurate insights into graft health and immune responses. This comprehensive approach is critical for the intricate care needed for VCA recipients to improve graft survival and function [4,120,121,148,149].

Recent progress in the field of transplant immunology has shifted the focus from immunosuppression towards immunomodulation, making it possible to successfully transplant vascularized composite allografts in the presence of new, less aggressive immunosuppressive regimens. In the long term, the goal is to induce donor-specific tolerance in transplantation representing the ultimate success in the field of transplantation: the acceptance of the allograft without the need to suppress the entire immune system and the burden represented by immunosuppressive therapy throughout the patient’s lifetime. However, this is still difficult to implement in a clinical setting. Current research is oriented towards achieving simple, safe, and durable protocols for inducing immune tolerance in the recipients of organ and vascularized composite transplants to extend accessibility to these therapeutic methods or at least to reduce the need for immunosuppressive therapy and implicitly decrease the side effect associated with it [21,22,23,150].

Belatacept, a unique FDA-approved co-stimulation inhibitor, offers a novel approach to immunosuppression in solid organ transplantation, particularly in kidney transplantation. Unlike traditional immunosuppressants that target T cells directly, belatacept inhibits T cell activation by blocking co-stimulatory signals necessary for their activation [41,125].

Approved specifically for the Epstein–Barr virus (EBV) seropositive kidney transplant recipients, belatacept is administered on a monthly basis as a weight-based intravenous infusion. Its long half-life of 8–9 days supports less frequent dosing, presenting an advantage for patients with compliance issues. Belatacept’s safety profile is notably favorable, with minimal nephrotoxic and metabolic effects, and does not require therapeutic drug monitoring. However, its use is cautioned against in patients with unknown or negative EBV serostatus due to an increased risk of post-transplant lymphoproliferative disorder [125].

The therapeutic value of belatacept extends to improving kidney function and metabolic outcomes, offering a viable alternative to calcineurin inhibitors (CNI) with a potential for reducing hypertension, diabetes and hyperlipidemia, with a potential use de novo in maintenance immunosuppressive therapy. Despite concerns over increased early rejection rates, evidence suggests that belatacept may reduce long-term mortality and allograft loss, albeit with nuanced recommendations for its use across different organ transplants [69,125].

In kidney transplantation, belatacept has shown promising results compared to tacrolimus-based maintenance immunosuppression regimens, improving kidney function but with an increase in rejection episodes that could be mitigated with transient tacrolimus use [125]. The de novo use of belatacept in liver transplant is not advisable due to the observed higher rates of mortality and graft loss [125].

Belatacept is also indicated as conversion therapy when the focus is on reducing or eliminating calcineurin inhibitors exposure due to their adverse reactions. In stable kidney transplant recipients, whether from living or deceased donors and identified as low immunologic risk, converting from calcineurin inhibitors to belatacept is considered safe. This transition has been associated with improvements in kidney allograft function and has been observed to moderately reduce the occurrence of new-onset diabetes and hypertension after transplantation. However, the potential advantages of this conversion need to be carefully balanced against the higher risks of acute rejection and infections, especially cytomegalovirus infection [125,151,152,153].

Also, in other organ transplant cases (liver, pancreas, intestine), where patients are dealing with adverse effects associated with CNI, particularly nephrotoxicity, considering a conversion to belatacept might be a viable option [125].

The impact of belatacept on donor-specific antibody (DSA) development and antibody-mediated rejection (AMR) also emerges as a critical area of interest. Preliminary evidence suggests a potential reduction in DSA incidence with belatacept use, pointing to its promising role in enhancing long-term transplant outcomes. Nonetheless, the full extent of belatacept’s effects on DSA and AMR remains to be further elucidated through ongoing research and clinical practice [125,154,155].

In the field of vascularized composite allotransplantation, existing research has also highlighted belatacept’s potential as an effective maintenance therapy.

Studies by Freitas et al. showed that in a cynomolgus monkey forearm VCA model, using belatacept in conjunction with tacrolimus led to enhanced graft longevity and inhibited the formation of donor-specific antibodies when compared with the combination of tacrolimus and steroids. This underlines belatacept’s role in extending the period without rejection when used alongside tacrolimus in VCA experimental settings [79,156].

Atia et al. evaluated belatacept’s efficacy when used alongside drugs that inhibit the Th17 immune response (such as ustekinumab and secukinumab) in a rhesus macaque VCA model and noted a quicker onset of acute rejection across all groups (within 14 days), compared to a historical cohort treated with conventional immunosuppression (tacrolimus, MMF and methylprednisolone), which had a mean survival of 31.1 days. Notably, this occurred regardless of Th17 response inhibition. Yet, in contrast to the control group without costimulation blockade, which saw a significant increase in DSA at the time of rejection, none of the belatacept-treated animals developed DSAs post-transplant [79,83].

Giannis et al. summarized the clinical experience using belatacept for vascularized composite allotransplantation, including six upper limb transplant recipients and one case of a face transplant. Six out of seven reported VCA patients (five hand transplant recipients and one male face transplant recipient) were converted to belatacept after developing an adverse reaction to their immunosuppressive therapy; the face transplant recipient converted to this therapy developed belatacept-resistant rejection (BRR) [79].

Cendales et al. reported the use of belatacept as de novo therapy in the initial maintenance phase for a unilateral hand allotransplanted male patient. After a successfully treated rejection episode, the maintenance immunosuppressive therapy for this patient was established as belatacept + MMF + prednisone and was reported as free of rejection [78].

**Figure 3:**
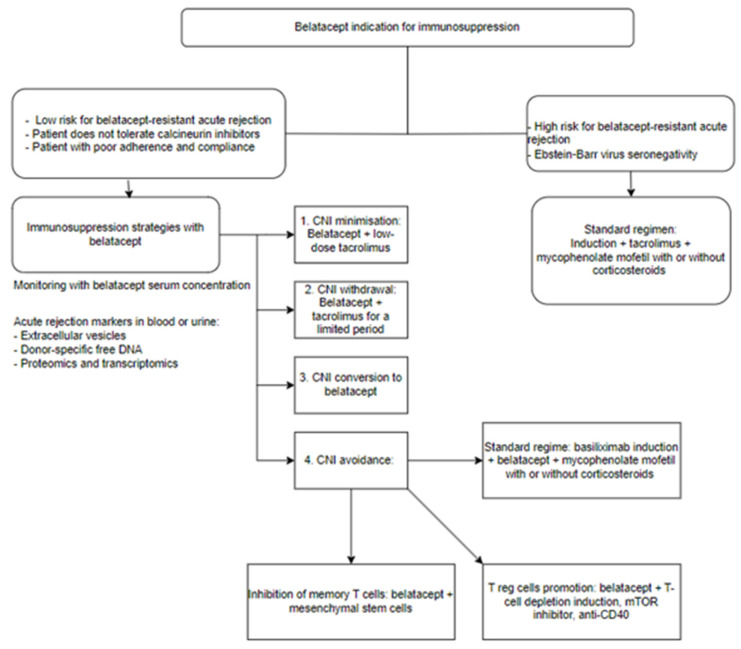
Proposed belatacept immunosuppression strategy, adapted after Marieke van der Zwan et al. [42].

Using biological agents to modulate the costimulation pathway is seen as a promising approach for preventing acute rejection, given its specificity compared to conventional pharmacological immunosuppression and its relatively limited non-immune toxicity, with belatacept currently being the only costimulation blockade therapy authorized for the prevention of transplant rejection. Future expansion of belatacept use might occur once specific indications and limits are established. A decisive immunosuppressive strategy using belatacept is presented in Figure 3 [42].

Utilizing PD-L1 as an immunomodulatory approach presents a potential strategy for preventing graft-versus-host disease (GVHD) as well as preventing transplant rejection. However, the function of this pathway in a clinical context remains to be precisely determined [87].

Immunotherapy has transformed the management of severe cancers, including cutaneous squamous cell carcinoma (CSCC). Cemiplimab, which acts as a PD-1 immune checkpoint inhibitor, has been approved by the FDA for treating locally advanced and metastatic cutaneous squamous cell carcinoma (CSCC) [157]. This type of tumor frequently arises as a complication in patients who are chronically immunosuppressed following a transplant. Cemiplimab represents a promising therapeutic option that can be incorporated into the treatment regimen for transplant patients requiring ongoing immunosuppression who develop this type of skin malignancy [87,158].

Bleselumab is a fully humanized immunoglobulin G4 monoclonal antibody targeting CD40. It suppresses both humoral and cellular immune responses by inhibiting the CD40–CD154 interaction among T cells, B cells, and antigen-presenting cells. It was introduced as a clinical strategy in kidney transplant patients, demonstrating non-inferiority to standard-of-care regimens when associated with tacrolimus, without significant immediate or long-term side effects. In these reports, transient liver enzymes as well as infectious risk with CMV and BK virus were increased, but without reports of thromboembolic events. For future implementation, a combination of costimulatory blockade with belatacept and an anti-CD40 monoclonal antibody molecule such as bleselumab may potentially induce a tolerogenic action [98,159].

The TIM gene family, a novel group of costimulatory molecules, is crucial in the activation and differentiation of Th cells. In animal models, the application of a blocking monoclonal antibody with a lower affinity for TIM-1 significantly extended the survival of completely mismatched cardiac allografts and, when used alongside rapamycin, induced tolerance. Human transplantation studies to date have predominantly explored Tim-3 as a marker for Th1 activation and rejection. Additionally, the measurement of TIM-3 and KIM-1 mRNA expressions, along with the detection of KIM-1 protein levels in urine and blood, shows promise as noninvasive methods for diagnosing allograft dysfunction [26,160].

Another targeted pathway for immunomodulatory purposes in transplantation is represented by the adhesion molecule blockade. LFA-1 antagonist efalizumab has been used in clinical regimens aimed at reducing the reliance on calcineurin inhibitors and steroids. It was employed in combination immunosuppressive therapies and succeeded in maintaining long-term insulin independence following one or two islet transplants of the pancreas. However, its use was discontinued due to the occurrence of progressive multifocal leukoencephalopathy in patients who received efalizumab. LFA-3 antagonist alefacept was used in the clinical setting of a kidney transplant but was associated with a higher malignancy rate [112,113,161].

Although there is not enough volume of clinical experience using costimulation blockade as an immunomodulatory strategy in solid organ transplantation as well as VCA, there may be advantages to the usage of such molecules on a larger scale in strategies that aim to decrease CNI usage, avoiding their significant side effects.

## 5. Conclusions

Over the past few decades, there has been a consistent increase in the utilization of human organs for transplantation, showcasing significant advancements in transplant medicine and its substantial therapeutic potential. Vascularized composite allotransplantation is a recent but very revolutionary transplantation branch which has achieved functional restoration and quality of life improvement in patients with extensive tissue defects, impossible to resolve with conventional surgical techniques. The major challenges in the VCA field are represented by immunological aspects, with the international community currently researching strategies that would minimize and optimize immunosuppressive therapy and, ideally, induce immunological tolerance, a situation difficult to achieve in clinical practice. Since its clinical emergence, the VCA field has benefited from immunological progress documented in wider organ transplantation programs.

Potentially tolerogenic therapeutic strategies are of great interest. Costimulatory blockade is one of the study directions explored in experimental models and further translated to clinical practice, based on promising therapeutic results.

Belatacept showed good results in recipients of solid organ transplantation in terms of allograft and patient survival and it can also be an elective therapeutic agent in clinical vascularized composite allotransplantation due to its immunosuppressive efficacy with fewer side effects compared to calcineurin inhibitors (an important aspect for VCA patients receiving transplant for quality of live improvement not as a life-saving procedure). An interesting approach is represented by the simultaneous blockade of multiple pathways of costimulation with synergistic effects in controlling T lymphocyte activation and preventing effector function.

The importance of costimulatory blockade will likely increase allowing a wider clinical use given the efficacy and safety of such agents in transplantation immunomodulatory responses. Future clinical trials comparing different costimulatory blockers will be necessary in order to understand their unique characteristics and benefits and elaborate comprehensive therapeutic protocols including these agents in immunologic therapeutic regimes.

## Figures and Tables

**Table 1:** A review of the costimulatory molecules classification according to their structure, in four major groups (families): [34,35,36,38,39,40,41,42,43,44,45,46,47,48,49].

	Group	Costimulatory Molecules on T Cell	Correspondent Ligands on the Antigen-Presenting Cell (APC)	Functional Attributes: Positive/Stimulatory Negative/Inhibitory
1	Immunoglobulin superfamily	CD 28 CTLA-4(CD152) ICOS PD1	CD 86 CD80 ICOS-L PDL-1/2	Stimulatory Inhibitory Stimulatory Inhibitory
2	TNF superfamily	CD40-L(CD154) CD27 CD30 OX40(CD 134) GITR 4-1 BB(CD137)	CD40 CD70 CD30-L OX40-L GITRL 4-1 BBL	Stimulatory
3	TIM family (T-cell immunoglobulin and mucin domain molecules)	TIM-1 TIM-3	TIM-4 Galectin-9	Stimulatory Inhibitory
4	Cell adhesion molecules	LFA-1 CD2	ICAM-1(CD54) LFA-3	Stimulatory

**Table 2:** Immunosuppressive agents used in face and upper limb transplant in induction and maintenance phases (%—frequency of usage) [131].

Phase of Immunosuppressive Therapy	Agents Used and Proportion of Cases Using the Therapeutic Agent	
	Face Transplant	Upper Limb Transplant
Induction Therapy	Thymoglobulin (88.9%). Methylprednisolone (73.3%) MMF (60.0%), Tacrolimus (53.3%), Prednisone (22.2%) Basiliximab (6.7%), Donor hematopoietic stem cell transplant (6.7%), Rituximab (4.4%), Extracorporeal photochemotherapy (2.2%), Anti-IL-2 mAb (2.2%), Alemtuzumab (2.2%).	Thymoglobulin (53.8%). Methylprednisolone (50.5%), MMF/MPA (35.2%), Tacrolimus (33.0%), Basiliximab (31.9%), Alemtuzumab (28.6%), Prednisone/Prednisolone/Steroids (26.4%). Mesenchymal stem cell transplant (12.1%), Cyclophosphamide (12.1%), Bone marrow cell infusion (3.3%)
Maintenance Therapy	Tacrolimus (97.8%) MMF (95.6%), Prednisolone/prednisone/steroids (73.3%) Methylprednisolone (24.4%) Extra-corporeal photopheresis (15.6%) Sirolimus (11.1%), Belatacept (2.2%), Everolimus (2.2%), Cyclosporine A (2.2%), Azathioprine (2.2%)	Tacrolimus (100%). Prednisone/Prednisolone/Steroids (94.5%) MMF/MPA (90.1%) Sirolimus (27.5%), Everolimus (9.9%), Belatacept (6.6%), Donor bone marrow infusion (5.5%).

## Data Availability

This study is a review. No new data were created.
